# Comparison between single-molecule and X-ray crystallography data on yeast F_1_-ATPase

**DOI:** 10.1038/srep08773

**Published:** 2015-03-10

**Authors:** Bradley C. Steel, Ashley L. Nord, Yamin Wang, Vijayakanth Pagadala, David M. Mueller, Richard M. Berry

**Affiliations:** 1 Department of Physics, University of Oxford, Clarendon Laboratory, Parks Road, Oxford, OX1 3PU UK; 2 Centre de Biochimie Structurale, 29 Rue de Navacelles, Montpellier, 34000, France; 3 Department of Biochemistry and Molecular Biology, Rosalind Franklin University of Medicine and Science, The Chicago Medical School, North Chicago, Illinois, 60064, USA

## Abstract

Single molecule studies in recent decades have elucidated the full chemo-mechanical cycle of F_1_-ATPase, mostly based on F_1_ from thermophilic bacteria. In contrast, high-resolution crystal structures are only available for mitochondrial F_1_. Here we present high resolution single molecule rotational data on F_1_ from *Saccharomyces cerevisiae*, obtained using new high throughput detection and analysis tools. Rotational data are presented for the wild type mitochondrial enzyme, a “liver” isoform, and six mutant forms of yeast F_1_ that have previously been demonstrated to be less efficient or partially uncoupled. The wild-type and “liver” isoforms show the same qualitative features as F_1_ from *Escherichia coli* and thermophilic bacteria. The analysis of the mutant forms revealed a delay at the catalytic dwell and associated decrease in V_max_, with magnitudes consistent with the level of disruption seen in the crystal structures. At least one of the mutant forms shows a previously un-observed dwell at the ATP binding angle, potentially attributable to slowed release of ADP. We discuss the correlation between crystal structures and single molecule results.

F_1_F_o_-ATP synthase is a rotary molecular complex responsible for the synthesis of ATP in bacteria and eukaryotes under aerobic conditions. ATP synthase couples energy from the electrochemical gradient across the cytoplasmic, mitochondrial, or chloroplast membrane, to the phosphorylation of ADP to ATP. The enzyme is comprised of two separable coaxial motors: F_1_ and F_o_. F_1_ is water-soluble and contains the catalytic sites used for the synthesis of ATP. F_o_ is membrane bound and acts as a proton turbine, using the electrochemical gradient to generate rotation that is physically coupled to F_1_ rotation. When isolated from F_o_, F_1_ behaves as an ATP-hydrolysing enzyme.

Figure 1A shows the atomic structure of yeast F_1_, and Figure 1B the mechanochemical cycle that couples ATP hydrolysis to rotation of the γ subunit in F_1_ from the thermophilic *Bacillus* PS3 (TF_1_)^1^ (for reviews, see Refs. 2,3,4,5). This enzyme's high functional stability and slow kinetics at room temperature facilitate extended single-molecule observation and resolution of mechano-chemical transitions. F_1_ from *E. coli* rotates 4–5 times faster at room temperature, with a similar mechanism^6^.

The TP, E and DP sites of the ground state crystal structure are believed to correspond to the states of subunits β_1_–β_3_ respectively at the 80° catalytic dwell, as illustrated in Fig. 1B^7^,^8^,^9^. All crystal structures to date have γ subunits within 20° of the ground state angle, most within 5°, indicating that all are variations on this ground state^10^. About half of these structures contain phosphate or an analogue at the E site, consistent with phosphate release at 80°. TP, E and DP states are best defined by their position with respect to the asymmetric γ subunit, as their nucleotide occupancy varies across different crystal structures. We label sites at the 0° dwell E*, TP*, and DP*, corresponding to the ground state they were most recently in if rotating in the ATP hydrolysis direction. A mechanochemical cycle can then be defined whereby a single β subunit in the E* conformation binds ATP at 0°, then moves through the TP and TP* conformations to hydrolyse ATP in the DP conformation at 200° and release ADP at 240° (DP*). Following rotation to 320° (E) it releases phosphate and is ready to bind ATP again at 360°^7^,^11^,^12^ The other two β subunits follow the same cycle delayed by 120° and 240°. TF_1_ and EF_1_ rotate unidirectionally but not continuously, stochastically entering a paused state due to Mg^2+^ ADP inhibition^13^.

Importantly, while most single molecule studies have been performed in TF_1_, the majority of high resolution crystal structures are for mitochondrial F_1_, with high resolution structures of bacterial F_1_ proving much harder to obtain^14^,^15^. Recent single-molecule studies of EF_1_^6^ have demonstrated the ability to resolve the kinetics of mesophilic F_1_ at room temperature. A very recent study of human F_1_ (HF_1_) reported that phosphate release and hydrolysis occur at 65° and 90° respectively after the previous ATP binding dwell, rather than both at 80° as in TF_1_ and EF_1_^16^. Single-molecule experiments on F_1_ from yeast will allow us to compare the mechanochemical cycles of different mitochondrial enzymes and to determine to what extent the extensive knowledge of TF_1_ can be applied to mitochondrial F_1_. It also allows the first comparison between single-molecule rotation studies and multiple high resolution crystal structures of F_1_ from the same organism. High-resolution X-ray crystal structures have been determined for bovine F_1_ in a number of different states, including a transition state with AlF_4_:ADP^17^,^18^,^19^,^20^,^21^ and for wild type yeast F_1_ in both the presence and absence of bound nucleotides^22^,^23^. For the yeast enzyme, the crystal lattice contained 3 complexes, with a well resolved phosphate bound to the E site in Complex II but absent in Complex I and III^22^.

In this paper, we investigate two classes of mutations to yeast F_1_: mitochondrial genome integrity mutations and the “liver” isoform. Mitochondrial genome integrity (*mgi*) is a class of mutations in the genes encoding the α, β, and γ subunits that allow petite negative yeast *K. lactis* to lose mitochondrial DNA^24^,^25^,^26^,^27^. The *mgi* mutations have been shown in yeast *S. cerevisiae* to uncouple proton flow from the synthesis of ATP^28^. While these mutations have been mapped exclusively to the α, β, and γ subunits of ATP synthase (Fig. 1A), the relationship between their effect on ATP synthase and the resulting yeast phenotypes is unclear. X-ray crystal structures have been determined for four *mgi* mutations^29^. These structures suggest two mechanisms for uncoupling and identify crucial regions involved. Mutations were grouped into two classes according to which mechanism is postulated: Class 1 mutations alter the structure of the E site and apparently disrupt phosphate binding, while Class 2 may disrupt interaction of the central stalk with the αβ pair^29^.

There are two isoforms of the γ subunit in humans and other mammals: a heart form and a liver form^30^. The heart form is expressed in heart, skeletal muscle, and intercostal muscle diaphragm, tissues of rapid and high-energy demand. The liver form is expressed in liver, cerebellar cortex, cerebrum, thyroid, spleen, pancreas, kidney and testis, tissues of relatively low or steady energy demand^30^. The isoforms are the result of alternative splicing. Isoform switching is regulated in a tissue specific manner and the alternative splicing can be reversibly induced by acid stimulation in muscle cells, but not in non-muscle cells^31^,^32^, suggesting that isoforms of the γ subunit in humans are a result of a carefully regulated alternate splicing process. This regulation is relevant to the physiology of the cell, yet the resulting effect on the biochemistry of the ATP synthase is unknown. The γ subunit expressed in the liver differs from that expressed in the heart only by the presence of an additional residue, Asp, at the C-terminus^30^. The tertiary structure of the yeast F_1_ is highly conserved with that of the bovine heart F_1_. Further, the primary sequence of the α and β subunit of the F_1_ ATPase is highly conserved from yeast to human. Based on the crystal structure of yeast and bovine F_1_ ATPase, it was predicted that the Asp at the C-terminus of the γ subunit could form a salt bridge with αArg288 in α_E_ (Pagadala V. and Mueller D.M., unpublished results, see SI for further details). If formed, the salt bridge is predicted to slow the catalytic cycle without decreasing the efficiency of the enzyme.

In this study, we use new high throughput detection and analysis tools to investigate the behaviour of wild type mitochondrial F_1_ from *S. cerevisiae* at varying ATP concentrations. We quantify the lifetimes of the ATP-binding and catalytic states, and present evidence that mitochondrial F_1_ rotation is governed by the same mechanism as that of the well-studied TF_1_ and the more recently studied EF_1_. We also use these tools to investigate the behaviour of seven forms of yeast F_1_ containing a point mutation, including six *mgi* mutations and a mutation that mimics the heart-to-liver isoform in humans. For four of these mutants, we discuss structure-function relationships with reference to high-resolution crystal structures.

## Results

### Yield of rotating gold beads

The rotation of yeast F_1_-ATPase was monitored by darkfield microscopy after attachment of gold particles to the γ subunit, as described in Methods. The position of all gold beads in the field of view was calculated and examined. A small fraction of beads showed clear circular trajectories (Fig. 2A), similar to those presented in previous work on F_1_. Many beads rotated unidirectionally as revealed by asymmetry in the power spectrum of their motion, but with signal-to-noise ratios too low for clear circular trajectories (Fig. 2B).

To assess the fraction of beads which were rotating, the directional ‘bias’ in rotation was examined (Methods). Rotation rate was assessed independently by fitting an ellipse to the bead trajectory and tracking the bead's angular speed around the ellipse - this accurately measures speed in high signal to noise conditions but underestimates speed when noise is significant. Fig. 2D-G shows bias vs. angular speed for F_1_-bead complexes at various ATP concentrations under identical imaging conditions. F_1_ is not expected to rotate in the absence of ATP, and under such conditions the plot is symmetric around the origin. When ATP is present, a ‘tail’ is visible in the direction corresponding to counter-clockwise rotation. Significantly, there is no corresponding tail in the direction that corresponds to clockwise rotation. This ATP dependent asymmetry indicates that beads with significant values of bias are rotating due to F_1_, even though many traces are too noisy to detect clear circular trajectories. In the ‘best samples,’ like those shown in Fig. 2, a conservative estimate is that at least 25% of the beads are rotating, and this is discussed further in SI.

The bias of a bead is related to the signal-to-noise value, which suggests that beads with a high bias should have high quality data. Qualitatively, this is observed. In work on bacterial F_1_, rotating molecules have typically been identified by eye, a laborious procedure subject to human selection. By measuring the bias of all recorded beads (potentially greater than 1000 per sample), the rotating molecules providing the highest quality data were quickly and automatically identified. In the SI, we discuss why a spread in bias/quality exists, and whether high quality spinners are representative of the broader population. In summary, data quality is principally determined by the orientation of the gold bead bound to F_1_ but is unlikely to significantly affect the kinetics. The spread in bias is not unique to F_1_ from yeast and is likely a common feature of all F_1_ single molecule spinning bead assays.

### Characterisation of wild type F_1_

Fig. 3 shows speeds measured for wild type yeast F_1_ as a function of ATP concentration using power spectra, and the more conventional angular rotation speed (Methods). Both fit well to Michaelis-Menten kinetics, with fit parameters of V_max_ = 586 ± 23 rev/s and K_m_ = 62 ± 9 μM, and 471 ± 79 rev/s and 59 ± 18 μM, respectively. Quoted errors correspond to the 95% confidence range of the fit. The difference between the two fits is primarily a result of selection bias in the smaller number of molecules used for the angular rotation speeds (see SI). Rotation speeds tended to be steady for individual molecules, but varied between molecules at the same ATP concentration, with a spread (sample deviation as fraction of mean) of 16% and 13% for the two methods when averaged over all ATP concentrations. The fit parameters are similar to those determined by biochemical methods (see SI). Biotinylation of the γ subunit caused no significant changes in the steady-state ATPase kinetic values.

Fig. 4B-D shows a single molecule displaying typical stepping patterns for low, intermediate and high ATP concentrations. At low and high ATP concentrations, three dwell positions are observed, while at intermediate ATP concentrations six dwells are visible. This is consistent with the model of F_1_ rotation developed using TF_1_, in which the ATP binding dwell, observed at low ATP concentrations, is located approximately 40° after the catalytic dwell, observed at high ATP concentrations^33^,^34^. By contrast, in HF_1_ six dwell positions were reported at high ATP concentrations^16^, indicating that HF_1_ is different from YF_1_ and TF_1_.

In contrast to reports from TF_1_, significant variation was observed in the measured angles between the binding and hydrolysis dwells. At intermediate ATP concentrations, typically either three broad peaks– likely due to unresolved closely separated dwells – or six peaks with separations above 40° were observed. To resolve closely separated peaks, flow cells were used to change the ATP concentration from 5 μM to 3 mM, and bead trajectories were analysed to compare the position of the dwells at low and high ATP concentration. Fig. 4A–D shows one such molecule captured before, during and after the flow of 3 mM ATP. Typically, dwell kinetics were assessed before and after the addition of 3 mM ATP (4B and 4D), see SI for details of the other molecules observed. Fig. 4E shows a histogram of the measured angles between the binding (5 μM) and hydrolysis (3 mM) dwells, as analysed using k-means clustering to identify the dwell locations at low and saturating ATP (Methods). A least squares Gaussian fit to the results from 18 molecules (54 dwell separations) gave a peak at 33° with a width of 19° (Fig. 4E). The median and mean separations were 31° and 34°. These measurements indicate that the separation between the binding and hydrolysis dwells in wild type yeast F_1_ is similar to the separation previously measured in TF_1_^34^ and EF_1_^6^, albeit the variation is larger.

The number of frames that rotating molecules spent in each dwell state for saturating (≥1 mM) and low (≤10 μM) concentrations of ATP were counted. A Bayesian information criterion (BIC)^35^ was used across models with 1, 2 or 3 sequential irreversible Poisson steps, both with and without a delay for bead rotation, to judge the quality of fit to each individual trace, as in previous work^6^. At saturating ATP, the data fit best using a short delay (0.13 ± 0.06 ms) and two ATP-independent steps (rate constants 2.5 ± 1.2 (ms)^−1^, 4.3 ± 2.3 (ms)^−1^). At low ATP (3–10 μM), the data fit best using one ATP-dependent step (18 ± 6 μM^−1^s^−1^) and one ATP-independent step with a rate (1.8 ± 1.2 (ms)^−1^) consistent with being an unresolved combination of the two steps fit at saturating ATP. Dwell distributions of a composite of 15 molecules at low and saturating ATP, and the best kinetic fits that result, are shown in Fig. 5. These results suggest that the rotation of wild type yeast F_1_ is governed by one ATP-dependent process located at the ATP binding dwell and two ATP-independent processes at the catalytic dwell.

Taken together, these results suggest that the fundamental kinetics of yeast F_1_ are similar to those of F_1_ from *E. coli* and the thermophilic *Bacillus* PS3, but different to those of human F_1_. That is, there is one ATP sensitive process located at the ATP dwell angle, then two ATP insensitive processes which occur following a 30–40° rotation of the central stalk. In addition, we observe a short delay at high ATP which is consistent with the expected time required for rotation of the gold bead following a mechanochemical step in F_1_.

### Analysis of “liver isoform” and six mutant forms

We used the methods developed here to screen seven forms of yeast F_1_ containing a single point mutation relative to the wild type enzyme. One mutant form corresponds to the “liver isoform”, while six forms contain mitochondrial genome integrity (*mgi*) mutations.

Fig. 6 shows speed-ATP curves for these mutant forms, and for comparison the wild type control from Fig. 3. The values associated with Michaelis Menten fits to each mutant are summarised in Table 1. Data obtained from the “liver isoform” mutant enzyme, γG278D, are indistinguishable from those of the wild type enzyme. This indicates that despite the tight regulation between the liver and heart isoforms, the Asp residue at the terminal end of the γ subunit has no measurable effect on kinetics of rotation under ATP hydrolysis. In contrast, all the data from the enzyme forms with *mgi* mutations show a significant reduction in the maximum rotation rate, by a factor of 2–8. However, the rotation rates of these mutant enzymes are only slightly affected at low ATP concentrations. There was a significant (at 95% confidence) decrease in the observed rate of ATP binding for the mutants βV279F, αN67I and αF405S, which had a binding rate approximately two thirds that of the wild type enzyme. Data of the mutant enzymes generally fit well to Michaelis Menten kinetics, with the exception of βR408I.

Kinetic analyses were performed on the dwell states for the six *mgi* mutant forms of the enzyme, as for the wild type, in order to determine the kinetics at low and saturating ATP. In all *mgi* mutant forms, the ATP binding dwell was best fit by a single exponential with an ATP-dependent rate constant assigned as k_ATP_. The catalytic dwell was best fit with two ATP-independent steps and an offset, similar to the wild type. The results are summarised in Table 1. Consistent with the Michaelis Menten fits (Fig. 6), the kinetics of the ATP binding dwell were changed relatively little, and those of the catalytic dwell much more, for all *mgi* mutants with respect to wild type. The rate of one of the processes occurring at the catalytic dwell dropped by as much as 10-fold, while the rate of the other process remained relatively unchanged. By comparison to TF_1_, we expect that these rates correspond to hydrolysis of ATP and release of phosphate, but this kinetic analysis does not determine which rate is altered, nor whether the same rate is altered in all mutants.

Data from the βR408I mutant showed an additional dwell at high ATP. At 3 mM, the rate of ATP binding is approximately 60 (ms)^−1^, which is too fast to be resolved in this study, and the expectation was this dwell would not be observed. Surprisingly, six dwells were clearly visible in the majority of high quality traces at both intermediate and saturating levels of ATP, as shown in Fig. 7A, despite the fact that k_ATP_ was not measurably different from wild type. Although these dwells were not typically observed at saturating ATP in the other forms of F_1_ investigated, a similar weak signal was occasionally present in traces from the wild type enzyme, as well as βR408G, αF405S and γI270T (Fig. 7B–E). However, due to the short duration in forms other than βR408I, and variability in its observation, we were unable to isolate and characterise this state. It is clear, however, that some process at the ATP binding angle was significantly slowed in βR408I even at saturating ATP concentrations. Accordingly, it is possible that a short, poorly resolvable ATP-insensitive dwell at the ATP binding angle may be a general feature of yeast mitochondrial F_1_ but amplified in the βR408I mutant form.

## Discussion

This study provides the first single molecule rotation analysis of F_1_ from a species where multiple x-ray crystal structures are also available, and the first comparison between high-resolution single molecule results from different mitochondrial F_1_s. In *S. cerevisiae* F_1_ as in bacterial F_1_, three ATP-independent processes occur approximately 33° before ATP binding. Given the similarity of our results to those with TF_1_ and EF_1_, we ascribe these to bead transit, ATP hydrolysis and phosphate release. This is identical to TF_1_, except that the equivalent work on TF_1_ lacked the time resolution necessary to observe bead transit^33^,^34^. By contrast, ATP hydrolysis and phosphate release in mitochondrial HF_1_ occur 30° and 55° before ATP binding, respectively^16^. It is possible that small differences between the angles of hydrolysis and phosphate release in YF_1_, TF_1_ and EF_1_ will be resolved by future improvements in temporal and spatial resolution^36^, but at present it appears that HF_1_ is different from the others in this respect. It remains to be seen whether these differences between different forms of F_1_ will show any discernible pattern; our results indicate that neither bacterial/mitochondrial nor thermophilic/mesophilc sources uniquely determine the pattern of catalytic dwell angles.

This study also demonstrates that the power spectral bias of gold bead position is a powerful method for quick, systematic detection of rotating molecules, facilitating the screening of several mutant forms. The power spectra can also be used to obtain speeds from noisy data traces, giving results comparable to those obtained using methods which require higher signal-to-noise.

The heart and liver isoforms of the γ subunit differ only by the addition of a single Asp residue at the C-terminus in the liver form, predicted to form a salt bridge with α_E_ that would slow the catalytic cycle. However, the results of the rotational studies here suggest this is not the mechanism of regulation, as the kinetics are unchanged. Instead, if the Asp serves in a regulatory mechanism, it likely occurs in conjunction with another molecule. The lack of observable effect of the C-terminal Asp is consistent with observations that crosslinking the equivalent residue to the α-subunit changes neither the catalytic activity nor the torque generated by ATP hydrolysis^37^,^38^.

Six *mgi* mutant forms of *S. cerevisiae* F_1_-ATPase were studied to investigate molecular defects which uncouple the yeast ATP synthase^28^. Mutations that largely or completely uncouple the enzyme are technically very difficult to work with; all of the *mgi* mutations studied here are “leaky”, meaning that significant coupling is still present. The most consistent and significant change across all mutants was a substantial slowing of one of the processes, presumably ATP hydrolysis or phosphate release, at the catalytic dwell^39^. Another clear feature of our results is that the ATP binding rates observed at limiting [ATP] were 70–100% of the wild type rate, while the catalytic rates at saturating [ATP] were 15–50% of the wild type rate. This indicates that these mutations strongly affect either the rate of ATP hydrolysis or the rate of phosphate release, but have negligible effect on ATP binding.

**Figure 1:**
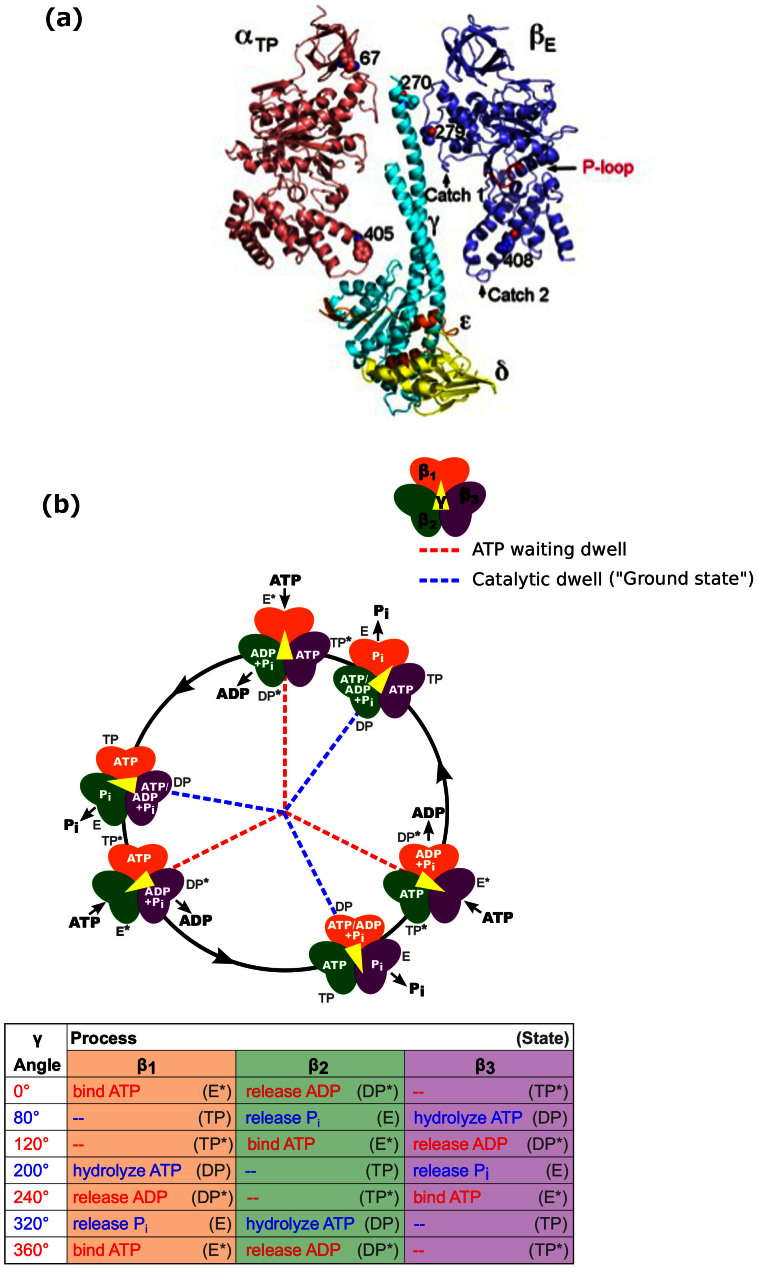
X-ray crystal structure and proposed mechanochemical cycle. (A) Locations of the *mgi* residues in the X-ray crystal structure of yeast F_1_ (pdb: 2HLD, Complex I, Ref. 22). The α_TP_, β_E_, γ, δ, and ε subunits are shown along with the residues that were mutated for this study. Also shown are the Catch 1 and 2 regions which interact with the γ subunit and help define the structure of the catalytic sites. The P-loop, which forms part of the P_i_ and ATP binding site, is coloured in red and labelled. (B) A proposed mechanochemical coupling scheme of thermophilic F_1_. The catalytic sites of the three β subunits are shown in orange (β_1_), green (β_2_), and purple (β_3_). We define the 0° binding dwell to correspond to ATP binding at a particular site labelled β_1_ and ADP release at β_2_^41^,^54^. The subunit shown in orange binds ATP at the 0° mark, and each subunit follows the processes and states listed in the table at the angles shown. The angular position of the γ subunit is represented by the central yellow arrow. Once a catalytic site binds an ATP molecule, it remains bound for 200° rotation of the γ subunit, whereupon it is hydrolysed. ADP is released at 240° and P_i_ at 320° from the binding site. Each β subunit completes one hydrolysis of an ATP molecule per 360° rotation of the γ subunit, and the three β subunits are staggered in their catalytic state by 120°. This means that at each ATP waiting dwell, ADP will release from one subunit and ATP will bind to the previous subunit. At each catalytic dwell, the ATP bound to one subunit will hydrolyze and the P_i_ from the previous subunit will be released. Refer to Refs. 2,3,4,5 for detailed reviews of the mechanochemical cycle of F_1_. Fig. redrawn from Ref. 2 with modifications.

*mgi* mutations have been categorized into two classes based upon their effect and location in the high-resolution structure of yeast F_1_ ATPase^29^. Class 1 mutations are located at the top of F_1_ (Fig. 1a) and form interactions with the E site at the Catch 1 region. The three Class 1 mutants studied here, γI270T, αN67I, and βV279F, showed no, minor, and significant conformational changes in the high-resolution crystal structure, respectively. Consistent with this pattern, the decrease in rotation speed was greatest for βV279F and smallest for γI270T. The changes to the high-resolution crystal structures were primarily around the E site, and the structure of the mutant forms αN67I and βV279F indicated that, unlike the wild type enzyme^29^, phosphate was not bound in the E site, suggesting that the affinity for phosphate has been reduced. Given the minimal disruption to the DP site in the crystal structure, it is probable that the slow rate observed for Class 1 mutants corresponds to phosphate release. The combination of structural and kinetic data therefore suggests that both the rate of phosphate release and the affinity for phosphate are reduced in the mutant. This implies that the binding rate of phosphate may be greatly reduced, consistent with impaired ATP synthesis.

The Class 2 mutations (αF405S, βR408I and βR408G) also show a significant reduction in the rate of one of the processes occurring at the catalytic dwell. Given the structural similarity of the E site to wild type in structural data, it seems likely that ATP hydrolysis at the DP site, rather than phosphate release, is slowed in Class 2 mutants.

αF405S, βR408I and βR408G are located at the bottom of F_1_, and while they are near the α/β interface, they do not change the conformation of the DP, TP or E active sites, but do alter the structure near the Catch 2 region. *mgi* residues αF405 and βR408 share a cation/π interaction^40^ and the crystal structure of αF405S shows a disruption in this interaction^29^. Both βR408G and βR408I also likely disrupt this interaction. The kinetic data for βR408I (and occasionally for βR408G and αF405S) showed an unexpected pattern of slow dwells at the ATP binding angle at saturating ATP. This slower dwell indicates a structural change in the conformation of F_1_ at the ATP binding dwell (in TP*, DP* or E*) and indicates a role for the cation/π interaction in one of the three reactions associated with the ATP binding dwell in bacterial F_1_; ATP binding, ADP release, or an unidentified temperature-sensitive step^7^,^33^,^41^. To discriminate between these three we note that the principal change in the crystal structure of αF405S is a disruption of the interactions between the γ and β subunits. Release of ADP occurs in the DP* state at or just after 240°, with rotation of the γ subunit greatly increasing the off-rate for ADP^42^. While ADP is being released from the DP* site, ATP is binding at the TP* site, and the two processes are co-ordinated by the γ subunit. We hypothesize that Class 2 mutations, as exemplified by αF405S, disrupt the αF405/βR408 cation/π interaction and interactions between the β and γ subunits that co-ordinate ATP binding and ADP release, and that the slowing of the binding dwell is due to the resulting decrease in the rate of ADP release.

Current crystal structures of F_1_ correspond to the catalytic dwell, and hence provide no direct structural information into the impact of Class 2 mutations on ADP release and the binding dwell. The speed of βR408I reduces with increasing [ATP] past K_m_, suggesting that conformational changes in the disrupted DP* site may be slowed and that ATP may weakly interact with the disrupted site. Alternatively, it is plausible that disruptions to coordination via the gamma subunit could delay the hydrolysis of ATP and allow rotation of F_1_ after phosphate release, but before ATP hydrolysis. The release of ADP would then be delayed until hydrolysis was complete. These predictions may be testable by computationally regenerating the ATP binding intermediate for Class 2 mutants.

Our single molecule data show no direct evidence of F_1_ moving into an uncoupled kinetic cycle where more than one ATP is consumed in a 120° rotation. However, the biochemical assays that show uncoupling involve situations where F_1_ experiences high load or is operated in the synthesis direction, and it is likely that these mutants remain tightly coupled when no load is present. Two technologies demonstrated on thermophilic F_1_ might be useful for investigating coupling – fluorescent ATP has been used to track ATP binding and ADP release from F_1_^43^, and magnetic handles have been used with magnetic tweezers to rotate F_1_ in the synthesis direction^44^, with one suggestion that uncoupling (“slips”) are observable in the wild type thermophilic enzyme^45^. Furthermore, we have not measured torque output from these mutants, which might correlate with coupling. We expect further work along these lines will help elucidate the mechanism of uncoupling in these mutants. Further, a crystallographic goal is to obtain the structure of F_1_ in a number of additional intermediate conformations including the ATP binding dwell conformation. The results in this study illustrate the power of single molecule analysis, the limitations of x-ray crystallographic data, and the complementarity of the approaches.

## Methods

### Covalent Modification of F_1_

Yeast F_1_ was genetically engineered using the QuikChange method (Agilent Tech.) to include two surface cysteine residues, γD101C and γE189C and a His_6_-tag on the amino end of the β-subunit, and was purified as described^46^. The cysteine residues were biotinylated by incubating: 10 mM (3.5 mg/ml) F_1_, 20 mM Biotin-PEAC5-maleimide (6-{N′-[2-(N-Maleimido)ethyl]-N-piperazinylamido}hexyl D-biotinamide, hydrochloride, Dojindo Laboratories), 50 mM HEPES, pH 8.0, 2 mM ATP, 2 mM MgSO_4_; for 30 min at 23°C, quenching with 100 mM DTT, and desalting on a centrifuge column containing Biogel P6 resin (Biorad). The protein concentration was adjusted to 0.4 mg/ml in buffer containing 50% glycerol, flash frozen and stored in liquid N_2_. This modification does not alter the activity of the enzyme.

### Gold bead preparation

60 nm gold beads (BBInternational) were functionalised with either Neutravidin or Streptavidin. Neutravidin beads were prepared by combining gold beads with 8 mg/mL Neutravidin in 10 mM Tris pH 8.0, and storing at 4°C for a minimum of 12 hours^47^. Streptavidin beads were prepared based on Ref. 48 by combining 5 μL each of 0.5 mM DSP, 10 mM TCEP and 100 μM streptavidin, each in 10 mM MOPS pH 7.0 with 50 mM KCl. This was incubated for five minutes, then 1 mL of gold beads was added as supplied, incubated for 2 hours at room temperature, and stored at 4°C. Beads were washed before use with excess 10 mM HEPES pH 7.0 (Neutravidin) or Tris pH 8.0 (streptavidin), followed by 10 mM MOPS pH 7.65, 50 mM KCl, 2 mM MgCl_2_.

### Rotation assays

Glass coverslips were functionalised with Ni^2+^ NTA as described previously^49^. Tunnel slides used double-sided tape to define a channel between a microscope slide and a Ni^2+^ NTA functionalised coverslip. Where the buffer was changed during microscopy, flow slides were used as described in SI, with two short tubes and the input tube connected to a syringe. F_1_ (1–10 nM in buffer A - 10 mM MOPS, 50 mM KCl, 2 mM MgCl_2_, 10 μM ATP, pH 7.65) and then gold beads (in buffer B = buffer A + 1 mg/mL BSA for streptavidin beads, or buffer A + 10 mg/mL BSA for neutravidin beads) were each added for ten minutes and washed with at least 5 volumes of buffer B. Rotation was observed in assay buffer (buffer B + ATP 1 μM–3 mM, 2 mM phosphoenolpyruvate and 20 units/mL pyruvate kinase). Where the concentration of ATP in the assay buffer was less than 10 μM, the ATP concentration of buffers A and B was reduced to the same level.

Tunnel slides were viewed under a high-speed backscattering darkfield microscope^50^ and images of multiple gold beads recorded at frame rates between 60 Hz and 30 kHz using a CMOS camera (Fastcam 1024 PCI, Photron) with 85 nm per pixel. For flow slides a custom stage with high mechanical stiffness replaced the piezo mount described in Ref. 50.

### Temperature

Microscope temperature was measured using a K-type thermocouple, and was 23.0 ± 0.5°C for all experiments used for speed or kinetic rate measurements. For flow slide experiments, used to measure the angle separation of substeps, the temperature was 22.5 ± 1.0°C.

### Video Analysis

Analysis of bead motion was performed using custom-written MATLAB software, as follows (see SI for details). Bead positions and intensities in each frame were calculated using a Gaussian mask algorithm^51^. Spinning beads were identified from directional bias in bead rotation,
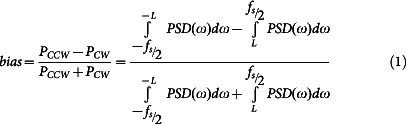
where *PSD*(ω) is the power spectral density at angular frequency ω of the bead's position (x(t), y(t)) expressed as the complex number z = x + iy, *f_S_* is the sampling rate and *L* is a low frequency cutoff used to remove noise associated with microscope drift, typically set to the lower of 5% of the typical rotation speed under the assay conditions used or 10 Hz. Beads identified as duplexes based on either radius of motion or intensity, were removed from the data set for all speed measurements and for kinetics measurements at high ATP (where the extra drag is significant). The software allows screening a sample to reliably detect rotating probes prior to acquisition of long videos.

### Rotation speed

**Figure 2:**
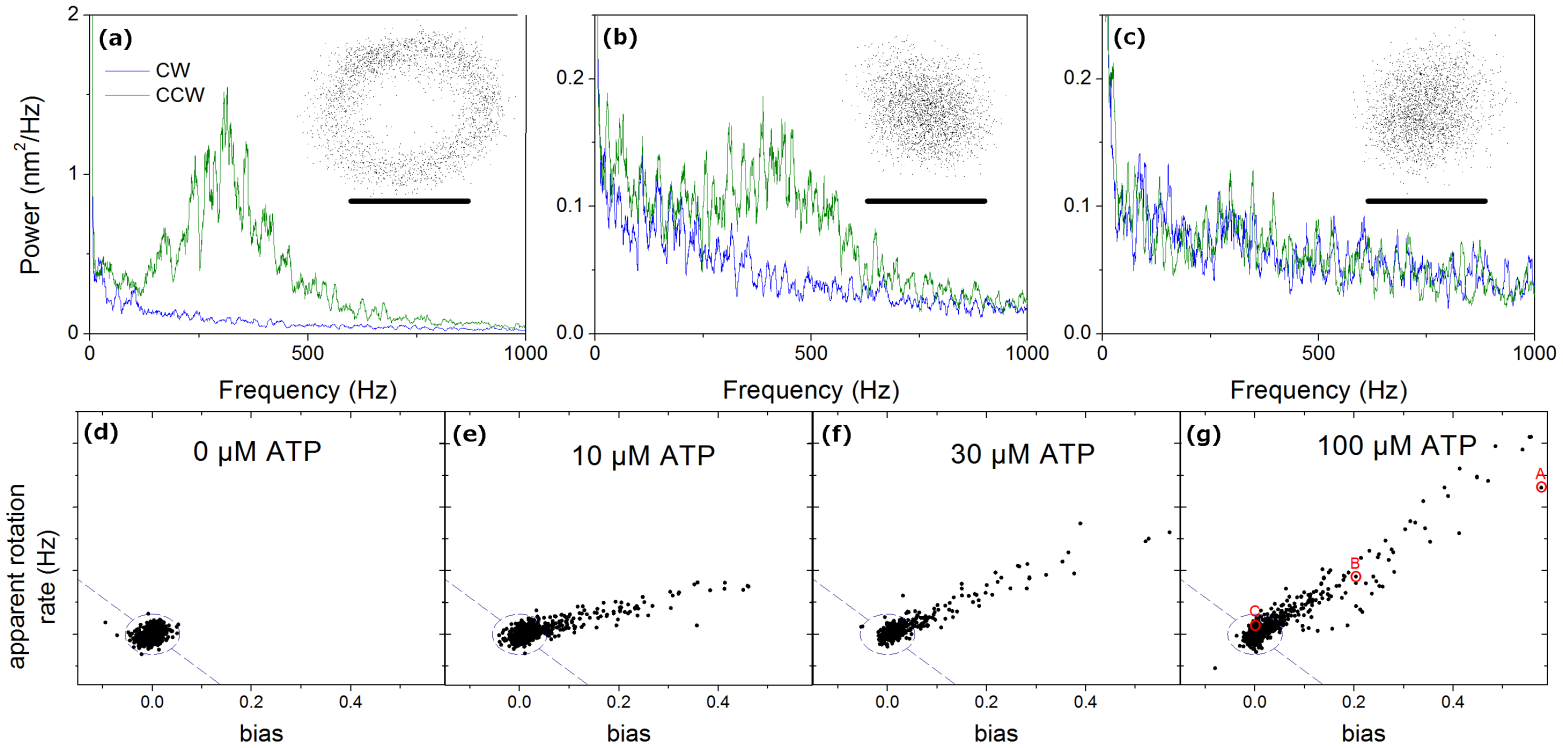
Example traces for F_1_ conjugated to neutravidin beads. (A–C) Bead trajectory (inset, scale bar 40 nm) and rotational power spectrum (counterclockwise, CCW, in green, clockwise, CW, in blue), for three representative molecules at 100 μM ATP. Trajectories show rotation clearly in (A) only, power spectra show clear evidence for a unidirectional rotation peak in both (A) and (B). (D–G) Power spectral bias against mean apparent angular speed for many molecules at different ATP concentrations. Without ATP, data are centred on (0, 0). With ATP, CCW rotating appear at positive speed and bias. The beads shown in (A–C) are marked with red circles. A 4-sigma ellipse is drawn based on the no-ATP data, and the external region split into CW rotation (lower left) and CCW rotation (upper right). 99.5% of the 0 μM ATP beads lie within this circle (955/960). When ATP is added, 21%, 28% and 27% of beads are on the counter-clockwise side of these thresholds at 10, 30 and 100 μM ATP respectively, with 0%, 0.3% and 0.2% on the clockwise side. The fraction of beads with bias above zero is 50.2%, 79%, 83% and 77% in (D–G) respectively.

**Figure 3:**
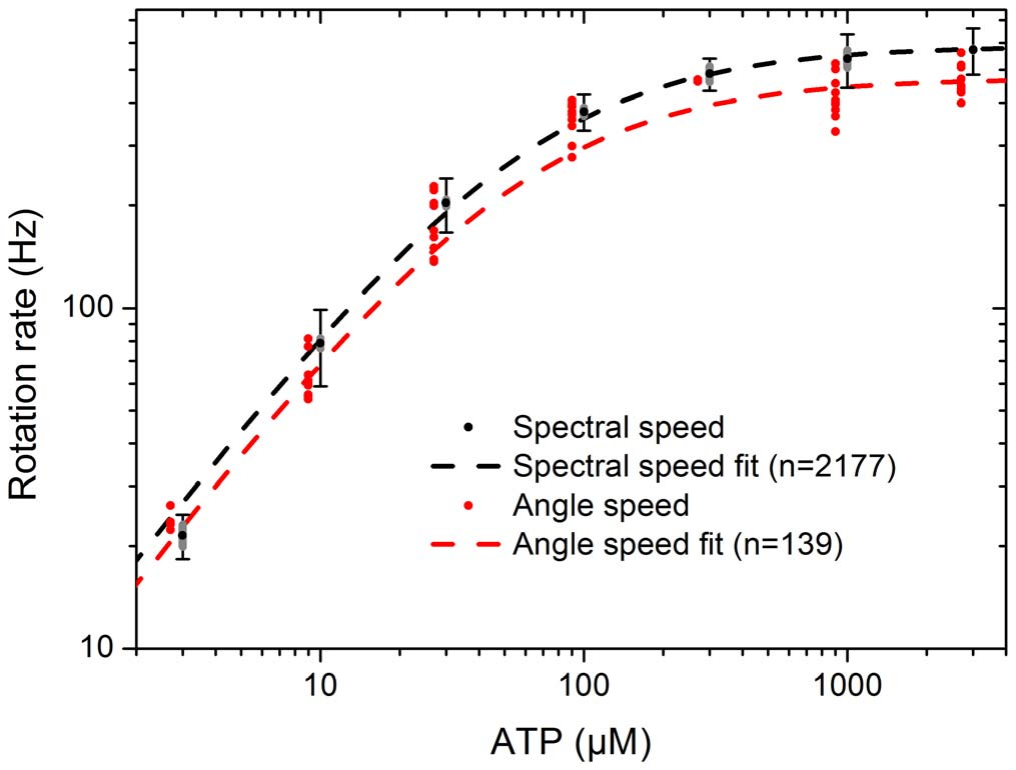
Rotation speed for wild type yeast F_1_ as a function of ATP concentration. Black points and fit line shows data from fitting the power spectra of 2177 beads. Error bars mark the sample deviation of speeds at each ATP concentration, and grey bars mark the 95% confidence interval on the mean. Red points and fit line show speeds of 139 beads selected by eye for trajectories such as that of Fig. 2A, determined from bead angles, points offset horizontally for clarity. The discrepancy between fits is due to selection bias for slow beads in angle speed data; see SI for comparisons. Michaelis-Menten fits were calculated using Origin software.

**Figure 4:**
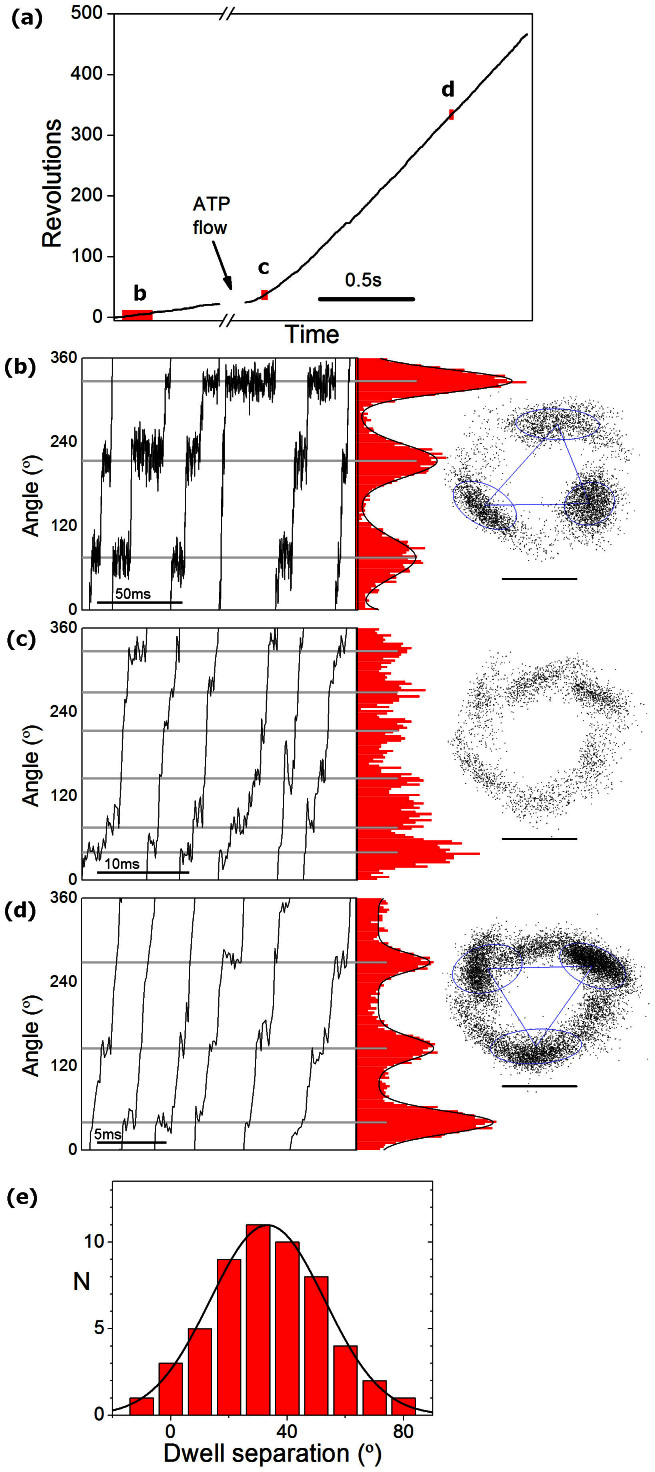
Dwells at low, intermediate, and saturating levels of ATP. (A–D) One F_1_ molecule observed at low, intermediate and saturating levels of ATP. (A) Unwrapped angle vs time for the full record of this molecule: after recording for 0.6 s at a low ATP concentration (~5 μM), 3 mM ATP was introduced to the flow slide and a further 1.5 s record collected. All data were collected at 9 kHz. (B–D) Angle vs time for a few revolutions at the locations marked in (A), and angle histograms (red) and trajectories (right, scale bar 40 nm) collected (B) prior to ATP flow, (C) in the first 0.3 s recorded after flow began, (D) in the remainder of the trace (once speed was stable). The precise ATP concentration at any moment at time is not known. This molecule was tagged with a gold duplex, giving a very clear rotation signal. Angular separation between the dwells was measured using k-means clustering to determine the location of dwells at low and high ATP, and measuring the angle change between the vectors connecting dwell locations (blue triangle on x-y plots). (E) Histogram of 54 dwell angle separations observed from 18 molecules and least squares Gaussian fit to the histogram. The molecule shown in (A–D) produced atypically large angular deviations (48, 49, 64°), compared to population median of 31°.

**Figure 5:**
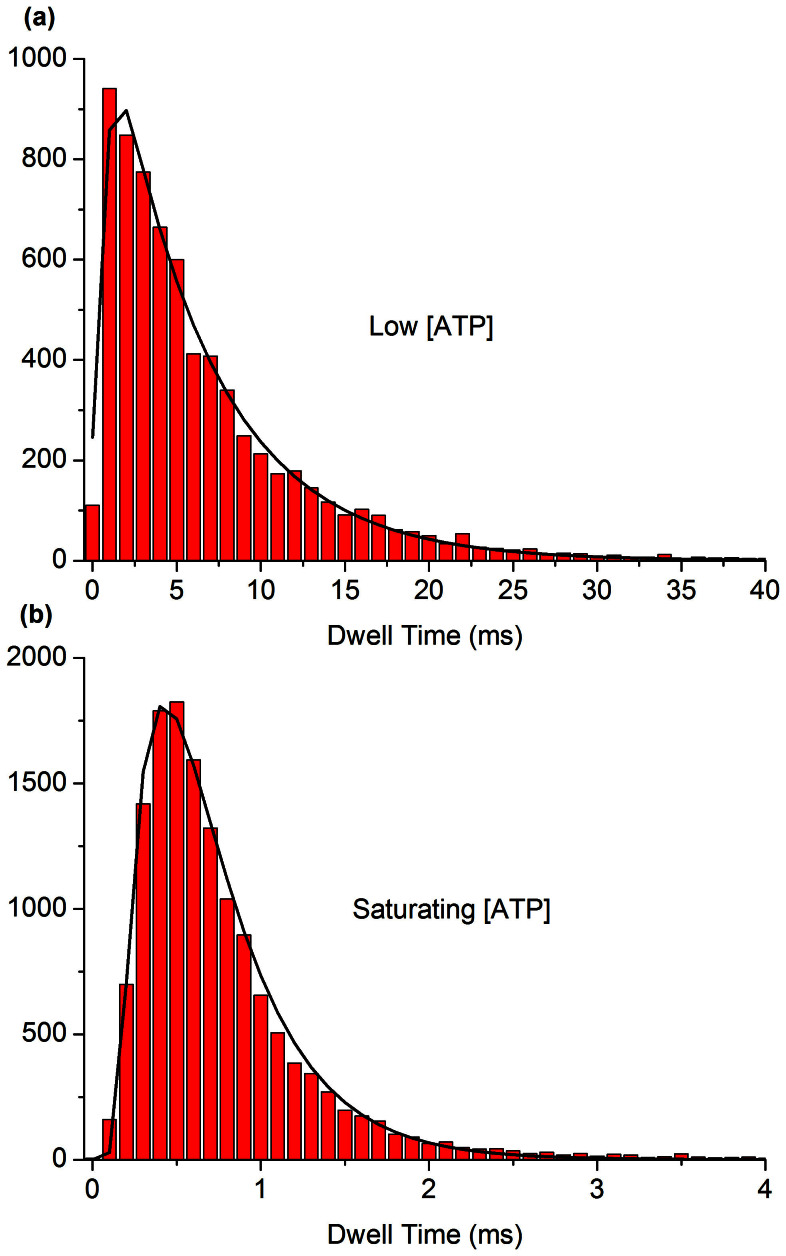
Dwell distributions for a composite of 15 wild type F_1_ molecules at (A) low (10 μM) ATP and (B) saturating (3 mM) ATP. The low ATP data are fit by two sequential Poisson processes, saturating ATP by two sequential Poisson processes with a time offset. The values for the kinetic rates and time offset from the fits of the composite dwell distributions agree with the kinetic rates and time offset calculated from the median values of single molecule fits within the 95 percent confidence intervals.

**Figure 6:**
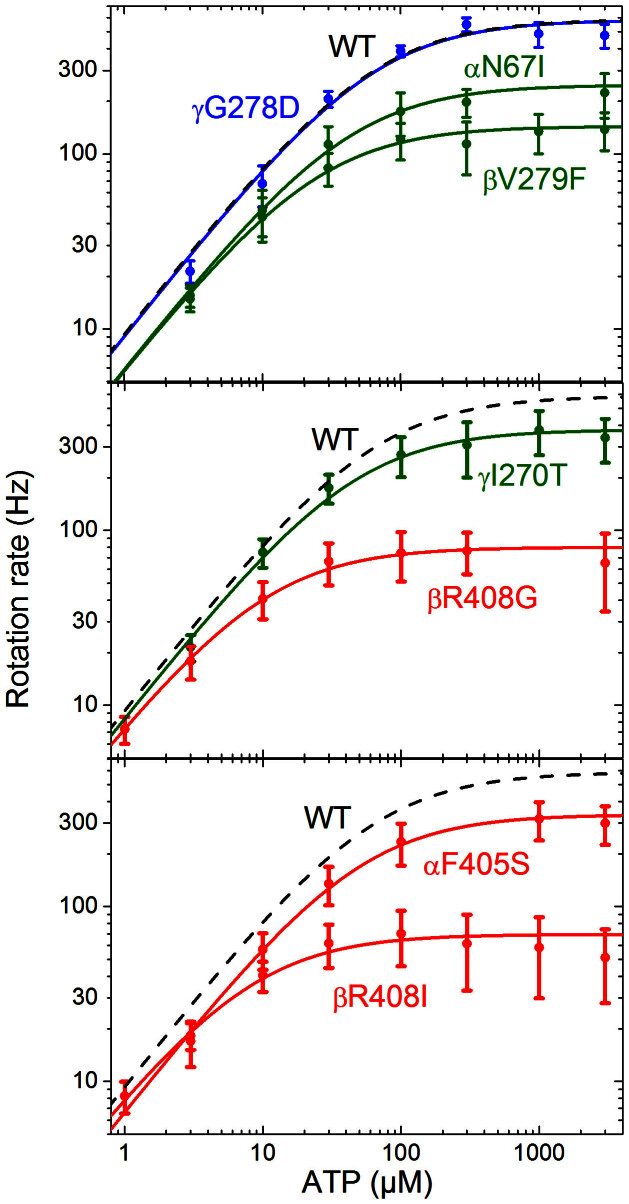
Rotation rate as a function of [ATP], for seven mutant forms of yeast F_1_, and associated Michaelis Menten fits (solid lines) for contrast to the wild type fit (grey dashed line) from Fig. 3. Class 1 mutations are shown in green, Class 2 mutations in red, with classes assigned according to crystal structures. Thin error bars mark the standard deviation of observed speeds; fat bars mark the 95% CI on the mean speed (in some cases these are too small to observe). Michaelis-Menten fits were calculated in Origin.

**Figure 7:**
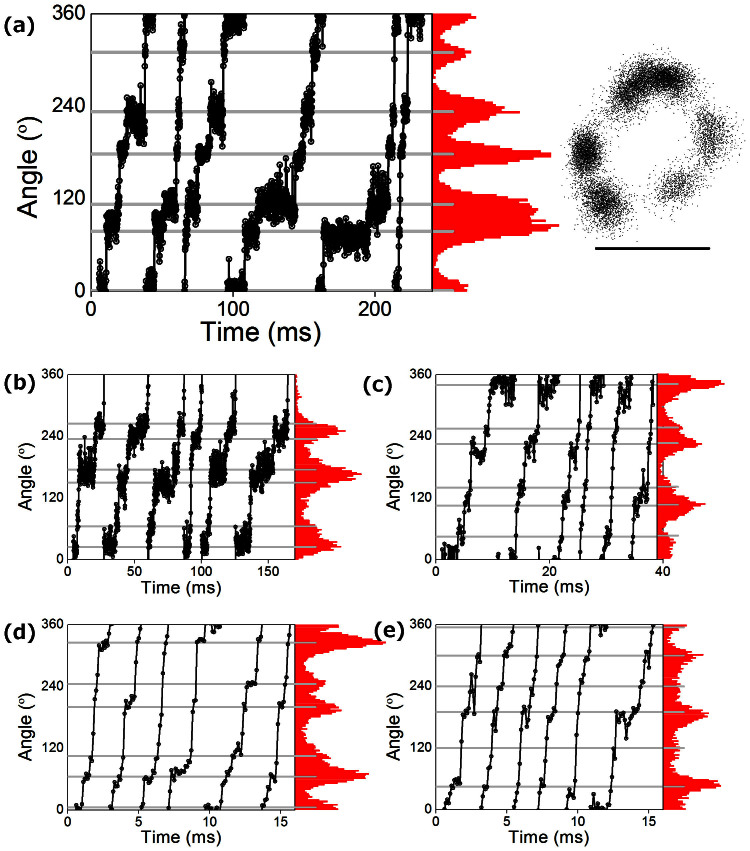
Six dwells at saturating ATP. (A) Six dwells are visible at saturating ATP for mutant βR408I. (B–E) Examples showing occasional dwells between major steps for (B) βR408G, (C) αF405S, (D) wild type and (E) γI270T. Note that while (A) is typical of data for βR408I, (substeps were detected by eye in only 1/21, 6/160, 3/45 and 5/360 high quality data traces for (B) βR408G, (C) αF405S, (D) wild type and (E) γI270T respectively. All traces shown were recorded at 10 kHz and 3 mM ATP, except (C) which was at 1 mM ATP. The expected ATP-dependent dwell is ~1/6 frame at 3 mM ATP, and would not be resolved. Scale bar marks 40 nm.

Ellipses were fit to the x-y data using an algebraic algorithm^52^, from which an unwrapped angular position was derived. Pauses, during which a bead stopped rotating, were identified as described below in the sub-section “kinetic fits”. Angular speed was estimated as the total angular displacement during the video divided by the total time, having first removed any pauses. Beads were excluded from speed measurements if more than 2% of the data points were closer to the centre of the fitted ellipse than one-third of the radius of the ellipse. Traces rejected by this criterion tend to have multiple false centre-crossings, and produce measured speeds that are lower than the actual rotation speed, as shown in Fig. 2.

Speed was also estimated as the peak in a Gaussian fit to the ‘subtracted’ power spectrum (*PSD*(−ω) - *PSD*(ω)), which isolates rotation in the CCW direction from non-rotational noise in the bead trajectory. If the trace contained pauses, data were restricted to the longest period for which an L_1_^53^ fit to the angle data indicated that the bead was rotating above one-third the maximum speed in the trace. Beads were excluded if they failed any of the following criteria: bias < 0.15 over the video, bias < 0.2 over the spinning period, the integral of the fit Gaussian <80% of the integral of the subtracted spectrum. A further symmetry criterion excluded data where the fitting mask moved between separate beads during the video (see SI for details).

### Substep angle measurements

Data were collected using a flow slide, observing the same field of molecules at low followed by saturating ATP concentrations. Comparison of videos collected before, during, and after buffer exchange, showed that there was typically translation of the field of view associated with flowing buffer through the slide, but no detectable rotation of the field of view.

For each molecule rotating before and after the buffer exchange, the last spinning episode before, and the first spinning episode after buffer exchange were selected. Episodes were discarded where low ATP speed was greater than 50 Hz or the final speed was less than 400 Hz (to ensure full buffer exchange), or the ratio of the long to short axis of the fit ellipse was greater than 1.5 (when angle recovery is less accurate). Measuring angles via ellipse fits before and after buffer exchange was unreliable, as a misplacement of the ellipse fit by a few nm significantly distorted the recovered angles. Instead, k-means clustering with 3 clusters was used to identify the dwell locations (x, y) at low and high ATP. The vectors between successive dwell states were determined, and the rotation of these vectors was used as the measurement of the angular displacement from the ATP hydrolysis to the ATP waiting state. This measures angle changes directly without requiring ellipse fitting or drift correction between the two sets of data.

### Kinetic fits

Kinetics were fit to angle-time traces in a manner similar to previously^6^. For fitting three dwells per revolution, the angle trace was unwrapped and broken into 120° segments, and the number of data points in each segment was used to generate a histogram of dwell times, pooling dwells from all 3 states. Pauses were removed by ignoring dwells longer than 7 times the mean dwell length. For single exponential kinetics this corresponds to removing the longest 0.1% of real (i.e., non-pause) dwells and a 0.6% (i.e. negligible) systematic underfit of the rate constants; for every other kinetic model the fraction of real events removed and effect on the fit is smaller. The dwells immediately preceding and following pauses were also removed.

A histogram of the dwell times was constructed for each enzyme. Six kinetic models were considered as potential fits to the histogram, comprised of 1, 2 or 3 kinetic rates per dwell both with and without a rotation-delay time offset. The dwell time histograms were fit to each model and rate constants were derived with a maximum likelihood estimation (MLE) algorithm. The likelihood of these six models and thus the quality of fit was compared using the Bayesian information criterion (BIC).

## Supplementary Material

Supplementary Information Supplementary Information

## Figures and Tables

**Table 1:** Dwell state kinetics

Form	Michaelis Menten fits				Dwell analysis			
	v_max_ (rev s^−1^)	K_m_ (μM)	k_ATP_ (s^−1^ μM^−1^)	n	k_ATP_ (s^−1^ μM^−1^)	k2 (ms)^−1^	k3 (ms)^−1^	n
Wild type	586 ± 24	62 ± 9	28.3 ± 3.6	2177	18 ± 6	2.5 ± 1.2	4.3 ± 2.3	18, 28
γG278D	582 ± 137	63 ± 33	27.6 ± 10.2	89	26 ± 2	2.6 ± 0.6	5.0 ± 1.7	4, 5
βV279F	144 ± 13	24 ± 5	17.9 ± 2.8	537	15 ± 2	0.36 ± 0.08	2.3 ± 2.7	4, 8
αN67I	248 ± 47	41 ± 12	18.0 ± 2.6	432	14 ± 3	0.46 ± 0.09	2.3 ± 0.7	7, 3
βR408G	80 ± 11	10.0 ± 2.6	24.1 ± 4.0	439	19 ± 5	0.21 ± 0.08	1.3 ± 0.5	10, 17
αF405S	336 ± 40	49 ± 11	20.4 ± 3.2	1044	16 ± 6	0.8 ± 0.6	2.0 ± 0.9	62, 40
γI270T	377 ± 92	44 ± 15	25.7 ± 4.4	2190	22 ± 7	0.8 ± 0.5	2.5 ± 2.0	109, 27
βR408I	69 ± 13	7.9 ± 2.5	26.4 ± 4.7	6519	23 ± 6	0.20 ± 0.07	3.3 ± 2.3	384, 51
*Bacillus* PS3^33^	129 ± 22	15 ± 5	25.8					
*E. coli*^6^	449 ± 39	21.1 ± 1.0	43 ± 4					

The results of the analysis from the Michaelis-Menten fits and the dwell time histogram analysis for wild type and the seven mutants screened. All values are quoted with 95% confidence intervals, with the exception of *Bacillus* PS3 and *E. coli*, where the values were sourced from previous studies as indicated in the table. The errors in these studies were typically quoted as standard deviations; for comparison to the 95% CIs listed here they have been multiplied by 1.96 for data where the number of degrees of freedom were unknown, or by the appropriate t-statistic where the number of degrees of freedom was known (2.45 to 1.96 for df = 6 to ∞). In some cases the error could not be calculated from the available data and has been left blank. The number of traces used is denoted by n. For the dwell analysis, the first value of n corresponds to the number of traces used to calculate k_ATP_ and the second value for the number of traces used to calculate k_2_ and k_3_.
