# Supplementary material for: Comparison between single-molecule and X-ray crystallography data on yeast F1-ATPase
Source: Sci Rep. 2015 Mar 10;5:8773. doi: 10.1038/srep08773 (PMC4894397; doi:10.1038/srep08773)
Supplement: Supplementary Information [file srep08773-s1.pdf]

Comparison between single-molecule and X-ray crystallography data on yeast F_1-ATPase.

Bradley C Steel[a], Ashley L Nord[ab], Yamin Wang[c], Vijayakanth Pagadala[c], David M Mueller[c], Richard M Berry[a*]

a Department of Physics, University of Oxford, Clarendon Laboratory, Parks Road, Oxford, OX1 3PU UK

b Centre de Biochimie Structurale, 29 Rue de Navacelles, Montpellier, 34000, France

c Department of Biochemistry and Molecular Biology, Rosalind Franklin University of Medicine and Science, The Chicago Medical School, North Chicago, Illinois, 60064, USA

* To whom correspondence should be addressed. Tel: 44 1865 272288; Email: r.berry1@physics.ox.ac.uk.

Supplementary Information

Figure Legends

Fig. S1: Structure of F_1 highlighting the position of the isoform difference between liver and heart F_1. (A) The α_E-subunit is shown on the left and g-subunit on the right of the arrow. The arrow indicates the location of the C-terminal residue, γ278Asp, which corresponds to the liver isoform, and α288Arg. (B) Enlarged view of the model showing the carboxylate group of γAsp278 and the guanidinum group of Arg288 in α_E.

Fig. S2: (A) Bias-speed plot for the sample analysed in (B-D). Beads were observed at 10 kHz for 0.87s; all data shown were from a single sample. Beads with high bias but low rotation speed are typically paused for a significant fraction of the observation. (B-D) Median (in blue) and upper and lower quartile ranges (in red) showing (B) radius, (C) noise and (D) speed, all assessed from the power spectrum, for beads pooled into bins spanning 0.05 in bias. Cyan lines show the total number of beads in each bin. Radius (Equation 1) was set to zero for negative biases rather than calculating the square root of a negative value. Dotted lines show a similar assessment with an intensity filter used to remove brighter beads that have a high probability of being double-bead complexes. Data with bias 0.2 or higher are a result of rotating F_1, at lower bias non-rotating beads are dominant. The radius of rotation is bias dependent to a maximum of close to one bead radius, while the noise levels are not. Above bias 0.2, rotation speed decreases slightly with radius.

Fig. S3: Rotation speed for wild type yeast F_1 as a function of ATP concentration using steady state experiments. Data points are shown in black with the Michaelis-Menten fit, calculated using Origin, shown in red.

Fig. S4: Molecules used for measuring the angles between substeps. Left: red denotes the xy-positions of the molecule at low ATP, and green the positions at high ATP. The location of the dwells, calculated using k-means clustering, is denoted with white (low ATP) and blue (high ATP) triangles. Centre: an angular histogram for each trace at 1º resolution (360 bins). Right: The histogram of dwell separations, as shown in Fig. 4e, with the three separations contributed by the molecule shown left/centre marked in red. The first bead shown is that presented in Fig. 4A-D.

Fig. S5: Side (left) and top (right) view of flow slide. Flow slides were constructed by drilling two holes through a slide and inserting plastic tubing. The tubing was held in place using epoxy resin. A tunnel slide was then constructed using a functionalized coverslip and double-sided tape, forming a channel between the slide and coverslip. Buffer was exchanged within the channel using gravity-flow by controlling the height of each end of plastic tubing.

Fig. S6: Comparison of Michaelis Menten speeds and kinetic data. The black points represent wildtype F_1 and the seven mutant forms screened, with error bars indicating 95% confidenc intervals. The dashed grey line represents the expected relation, an agreement between the two methods.

Figures

Fig. S1

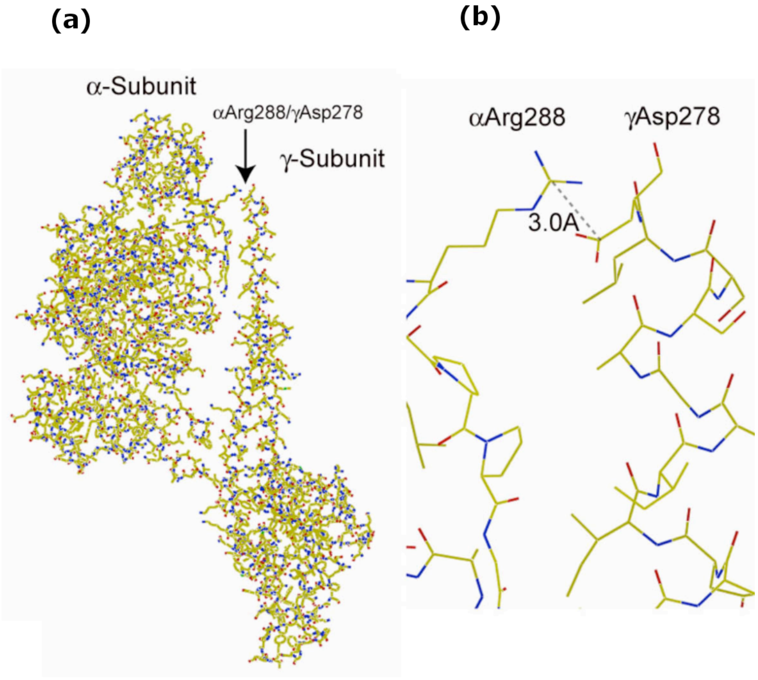

Fig. S2

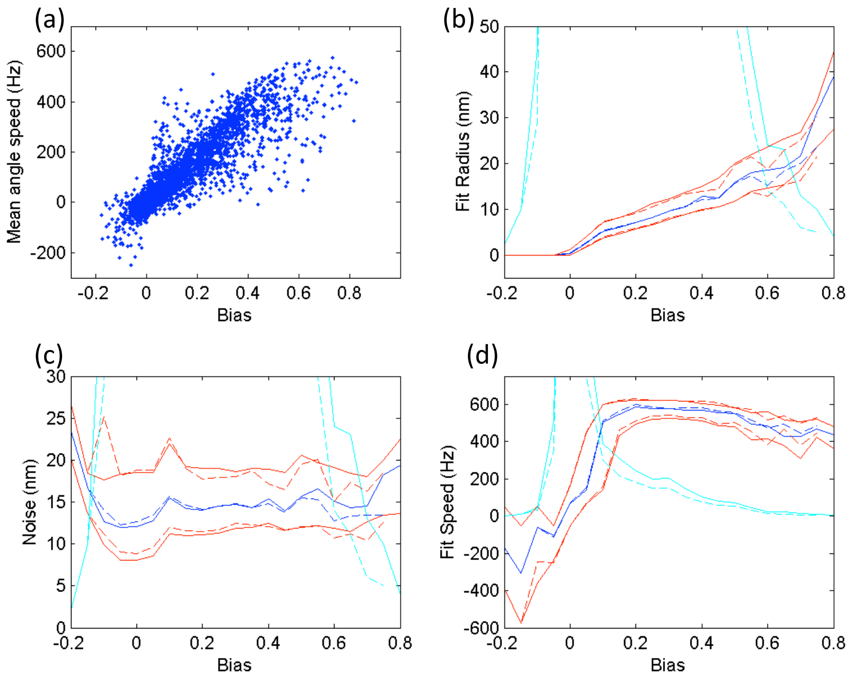

(a)
Mean angle speed (Hz)
600
400
200
0
-200
-0.2
0
0.2
0.4
0.6
0.8
Bias
(b)
Fit Radius (nm)
50
40
30
20
10
0
Bias
(c)
Noise (nm)
30
25
20
15
10
5
0
Bias
(d)
Fit Speed (Hz)
600
400
200
0
-200
-400
-600
Bias

Fig. S3

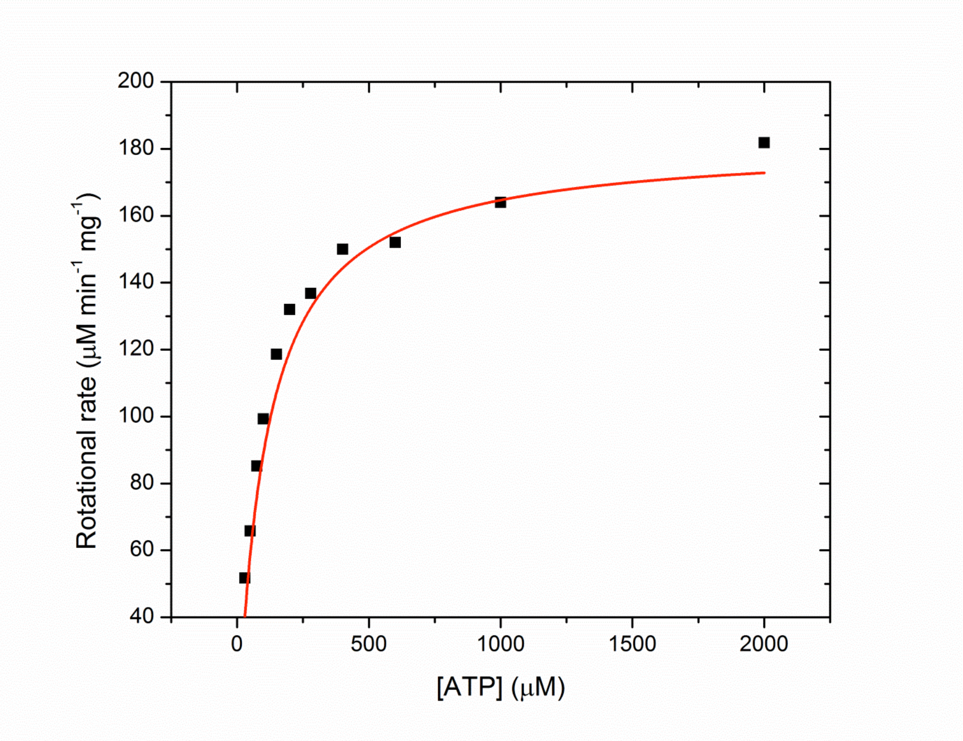

Rotational rate (μM min-1 mg-1)
200
180
160
140
120
100
80
60
40
0
500
1000
1500
2000
[ATP] (μM)

Fig. S4

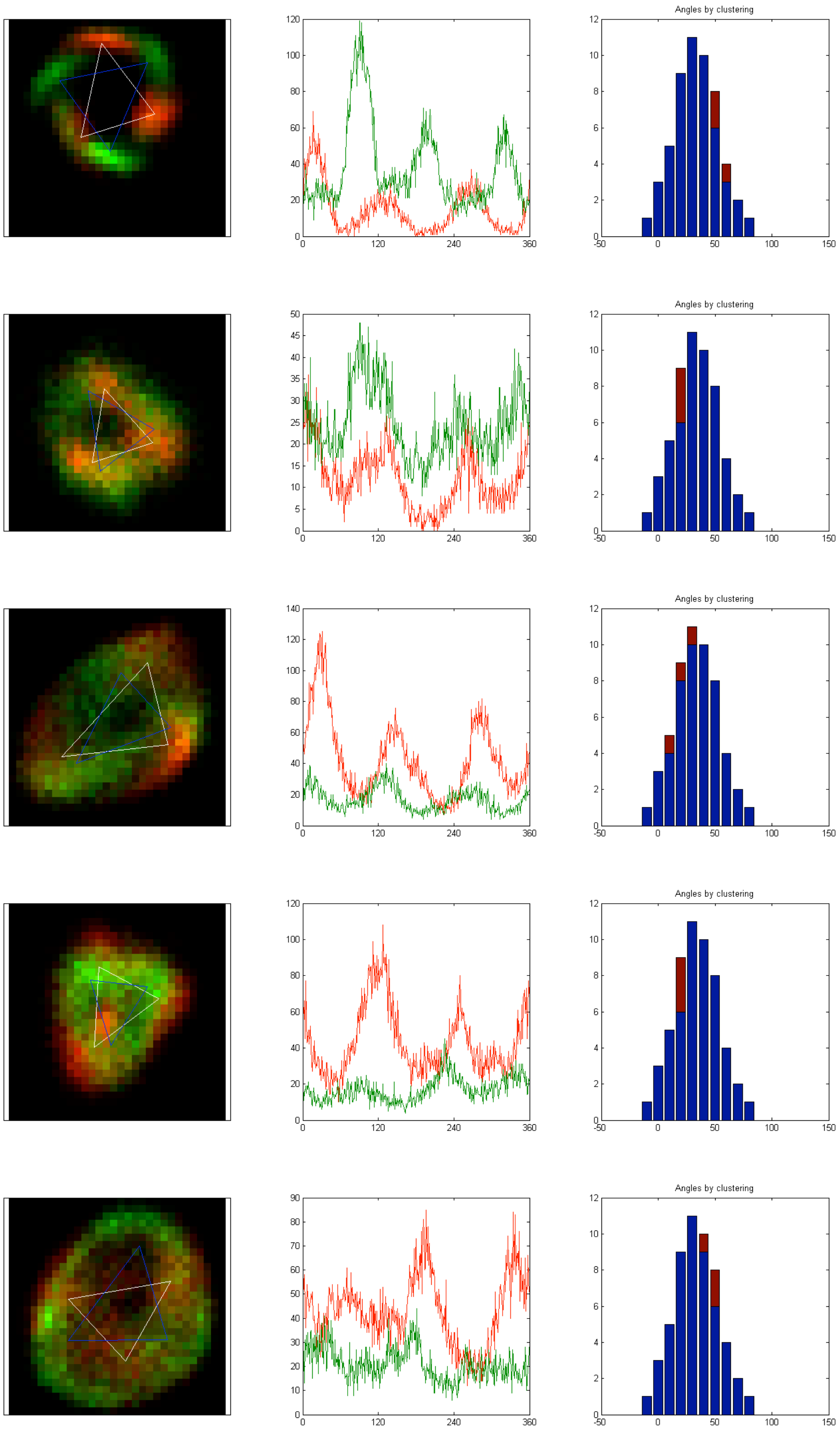

Angles by clustering
Angles by clustering
Angles by clustering
Angles by clustering
Angles by clustering

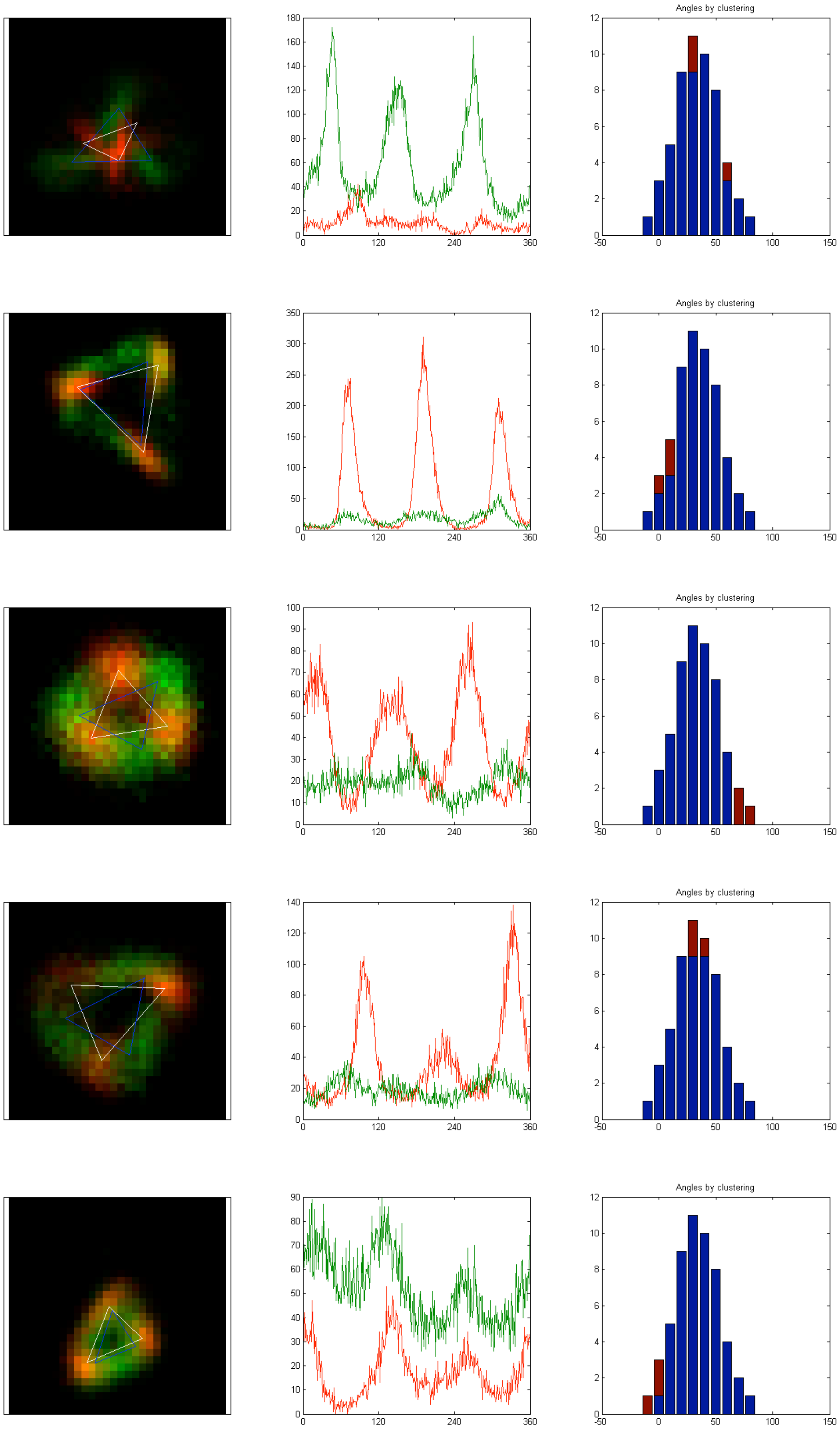

Angles by clustering
Angles by clustering
Angles by clustering
Angles by clustering
Angles by clustering
0 120 240 360
-50 0 50 100 150

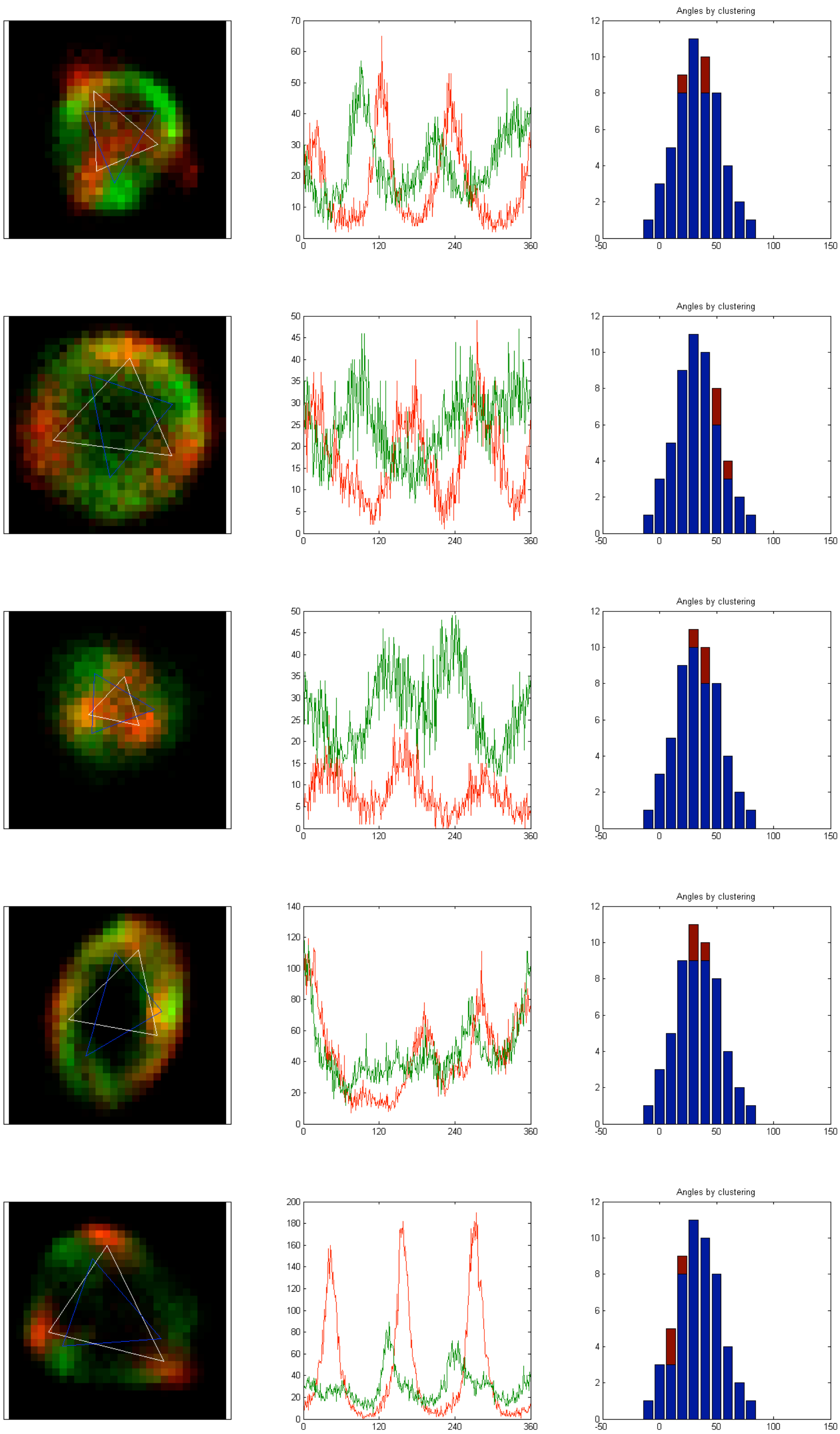
Angles by clustering
Angles by clustering
Angles by clustering
Angles by clustering
Angles by clustering

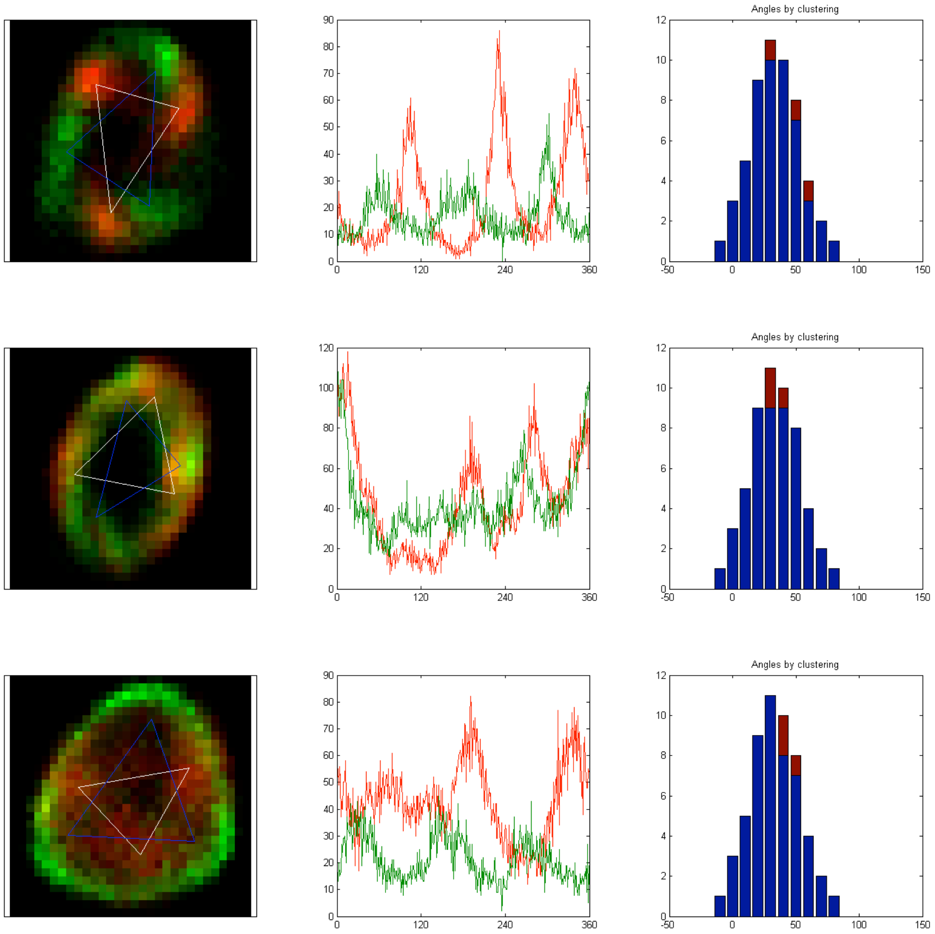
Angles by clustering
Angles by clustering
Angles by clustering

Fig. S5

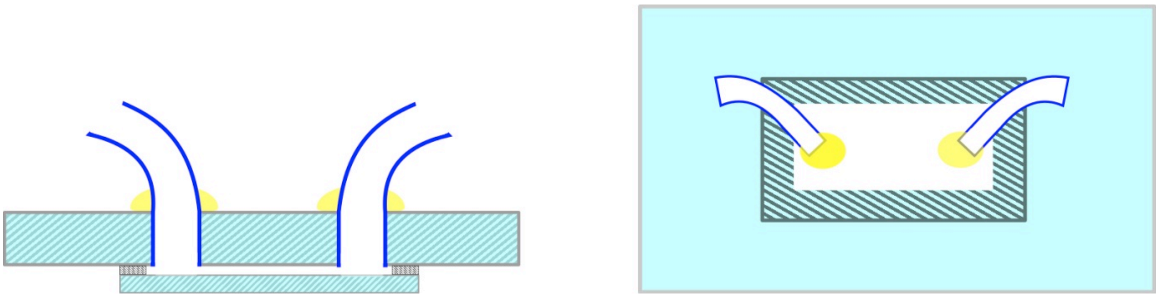

Fig. S6

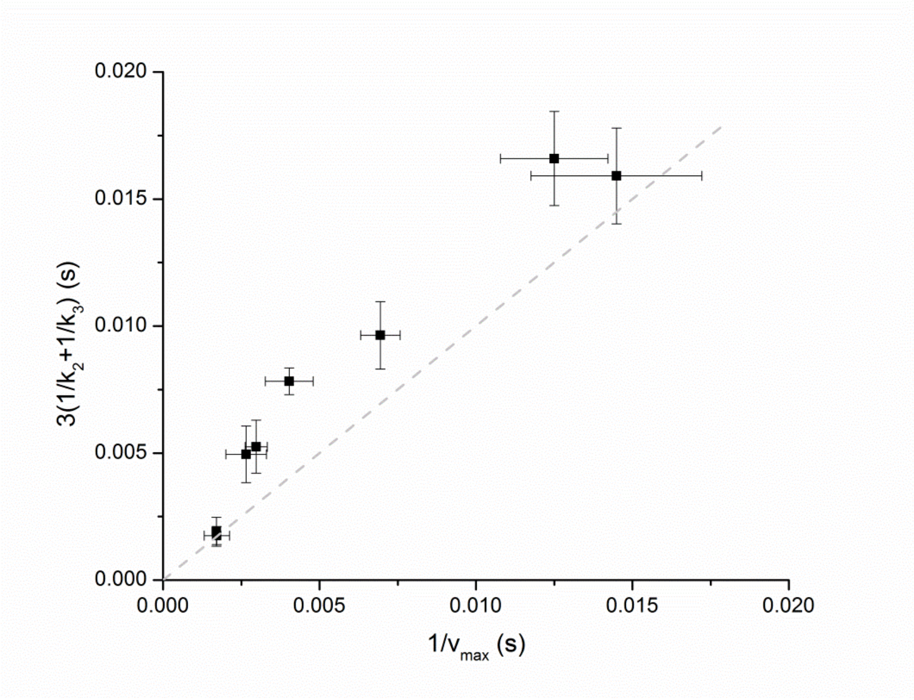

Supplementary Information Text

Yield of spinning beads

Using an elliptical 4-sigma threshold based on the no-ATP data to identify non-spinners, a conservative estimate is that at least 25% of beads are rotating in the 'best' samples, like those of Fig. 2. This fraction varied between 4% and 30% across all assays (typically ~ 10%), suggesting variability in assay preparation and quality of the F_1 samples are significant in final yield, although acquisition conditions also affect these values. An unbiased, albeit noisy, estimation of the rotating fraction can be obtained by counting the number of beads with counter-clockwise bias and subtracting the number with clockwise bias. This estimated that as many as 60 % of the molecules shown in Fig. 2 were measurably rotating. These calculations are based on videos that are 2.6 seconds long. F_1 is known to have a 'paused' state, and any enzyme, which remains paused throughout the video will not be measured to rotate.

Predominance of low-bias spinners

As shown in Fig. 2, the majority of rotating beads have low bias and hence low data-quality. One obvious question – since the vast majority of the rotating molecules do not have high bias or high quality data, and would not be selected in a search by eye – is whether those with high bias are representative of the larger population. This is not a question unique to yeast F_1; analysis of videos of *E. coli* F_1 conjugated to 40 or 60 nm gold beads, collected both by ourselves and by the Futai laboratory, show identical patterns on a speed-bias plot, making it likely that this is common feature of all F_1 low-load single molecule experiments that are based on tracking a rotating spherical probe (note that experiments which typically track duplex beads may have a very different distribution on such a plot). We have taken two approaches to answering this question; the first investigates why such a large range of biases exists, and the second looks at the rotation speed of molecules as a function of bias.

Firstly, molecules with lower bias may fall into this category for two basic reasons – the power spectral magnitude of their rotation may be smaller, or the power spectral magnitude

of noise associated with the measurement may be greater. We estimate these values by making the approximation that rotation appears only on the counter-clockwise side of the power-spectrum, while noise appears symmetrically on both sides of the power spectrum, to give two parameters associated with the 'signal' and the 'noise':

$$Signal \cong P_{CCW} - P_{CW} \quad (1)$$

$$Noise \cong P_{CW} \quad (2)$$

The square root of these values has units of nanometres, and represents the radius of the bead motion (signal) or the measurement noise per axis (dominated by Brownian motion). In the case of elliptical motion, the signal is proportional only to the short axis of the ellipse, and some extra signal will leak into the noise term. A plot of these parameters as a function of bias is shown in Fig. S2A-B, and clearly indicates that high quality data is a function of large rotation radius rather than lower noise imaging.

The rotational speed obtained by measuring the angular position of a bead is strongly noise dependent – when a trace forms a clear circular pattern it is accurate, but once noise dominates, forward rotations are often misidentified as backward rotations, underestimating the speed. Instead, we used the location of peaks in the power spectrum to estimate the speed of rotating beads. This is common practise in other systems, for example for measuring the rotational speed of the bacterial flagellar motor [1], and is a measurement that is insensitive to noise. For F_1, the time taken for each revolution varies in a stochastic manner governed by the underlying kinetic rates, with a significantly wider distribution of times than is seen in the bacterial flagellar motor. This corresponds to a wide, rather than sharp, peak in the power spectrum of a rotating bead, at a location close to that which would be measured by tracking rotation. The ratio of these speeds typically tends to 1 for high quality data, except for very low ATP conditions. We used the peak of a Gaussian fit to the power spectrum as an estimate of the speed of rotating beads. As shown in Fig. S2C, there was a small reduction in speed as a function of bias for conditions of saturating ATP. We suggest that this difference is most likely due to the slight increase in drag associated with rotating a 60 nm gold bead at non-zero radius.

These results suggest that the 'high quality' beads assessed in this and previous low-load studies of F_1 are of a subpopulation where the bead is linked to F_1 in a manner that produces a large radius of rotation when F_1 rotates. Nonetheless, in the absence of contrary evidence, we assume, as previous studies have done, that the underlying kinetics of the enzyme are not correlated to the orientation with which the bead links to the enzyme, other than as is needed to provide additional torque to rotate the bead. The effect of this is small; a standard drag calculation, using DLS measurements for the beads' hydrodynamic drag coefficient and a typical torque estimate of 40 pN.nm, suggests the delay due to drag should be about 0.2 ms per revolution.

Comparison with biochemical methods

Steady state methods, under apparent tri-state conditions and assuming 3 ATPs hydrolysed per rotation, yield kinetic values V_{max} = 182 mmoles/min/mg (364Hz) and K_m = 85 mM. These kinetic values are similar to those determined using power spectra and angular rotation speed. The data from steady state analysis is shown in Fig. S3.

Molecules used for substep angle measurement

Analysis of the molecules used for measuring substep angles are shown in Fig. S4.

Flow slide

For experiments where the buffer was changed during microscopy, flow slides were constructed as shown in Fig. S5.

Video Analysis

Analysis of bead motion was performed using custom-written MATLAB software, which can be broken up into a number of discrete steps.

Firstly, gold beads were identified by looking for local maxima after high pass (1200 nm) and low pass (150 nm) spatial filtering of an average of the first ten video frames. The position of the bead in each frame was calculated using the Gaussian mask algorithm of Thompson (1), initially seeded using the centroid and subsequently the average position over the preceding 100 frames, evaluated over a square region of interest 1.2 µm on each side and centred on the initial position of the gold bead. Bead intensities were estimated for each frame by summing the pixel intensities after the mask had been applied to the image and normalising by the product of the mask with itself. The position and intensity measures obtained this way are identical to those produced by a fixed width least squares Gaussian fit, but the Gaussian mask procedure is computationally faster.

Secondly, spinning beads were identified and data from other beads (non-spinning or not easily categorised) were discarded. To define these classifications, we calculated the power spectrum of each bead's position (x,y), expressed as z=x+iy, and compared the magnitudes at positive and negative frequencies. We define the 'bias' in the power spectrum as

$$\mathbf{bias} = \frac{\mathbf{P_{CCW}} - \mathbf{P_{CW}}}{\mathbf{P_{CCW}} + \mathbf{P_{CW}}} = \frac{\int_{-f_S/2}^{-L} \mathbf{PSD}(\omega)\mathbf{d}\omega - \int_{L}^{f_S/2} \mathbf{PSD}(\omega)\mathbf{d}\omega}{\int_{-f_S/2}^{-L} \mathbf{PSD}(\omega)\mathbf{d}\omega + \int_{L}^{f_S/2} \mathbf{PSD}(\omega)\mathbf{d}\omega} \tag{3}$$

where *PSD*(w) is the power spectral density at a given frequency, (rotation direction is defined for viewing from the gamma subunit side of F_1 as is standard in F_1 literature, although typically we view from the opposite direction), f_S is the sampling rate and *L* is a low frequency cutoff used to remove noise associated with microscope drift. *L* was typically set to the lower of 10 Hz or 5% of the typical rotation speed under the assay conditions used.

As discussed in the results, beads with low values of bias were not considered to be measurably rotating. Appropriate thresholds (≥ 0.15) depend on acquisition settings. Beads with high values of bias were checked manually and were typically spinning, although rare artefacts arise due to mis-tracking in the Gaussian mask algorithm (identified by large, near-instantaneous jumps in the bead position) or, very rarely (ie, < 0.1% of videos), rotary resonance modes excited in the microscope's piezo stage and common to all beads in that video. Beads which appeared to be unresolved duplexes, based on either a high radius of motion or high bead intensity, were removed from the data set for all speed measurements and for kinetics measurements at high ATP (where the extra drag is expected to affect the results).

The MATLAB software we developed is available from the authors and will be made freely available on the MATLAB File Exchange following publication. On an Optiplex 780 (Core 2 Quad Q9400, 2008, 4GB RAM, Windows 7), calculation of bead position proceeds at approximately 10^5 positions per second using the Gaussian mask routine. Total analysis time, including video read and data analysis, depends on video properties but is typically 1-5 × 10^4 positions/s.

Our camera is not capable of continuous acquisition and recorded data must be downloaded from camera memory. The software is capable of automatically analysing videos upon download; for a 256x160 pixel field of view at normal densities (<400 particles), analysis of 1000 frames can be done in < 10 seconds, followed by display of data traces from rotating particles. These algorithms potentially allow screening a sample to reliably detect rotating probes prior to acquisition of long videos.

Rotation speed

Ellipses were fit to the x-y data using an algebraic algorithm (2), from which an angular position was derived. Angle was 'unwrapped' by assuming that the bead never rotated more than π radians between successive frames. Two measures of the speed of rotation were calculated for each bead. First, the power spectrum of the bead's motion was calculated over both the full trace and, if the trace contained pauses, over the longest period for which an L_1 (3) fit to the angle data indicated that the bead was rotating at a minimum of one-third the maximum fit speed in the trace. The 'subtracted' power spectrum was calculated by subtracting the CW side of the power spectrum from the CCW side, and the resulting trace (which should show only rotational motion plus noise) was fit to a Gaussian function. The peak of the Gaussian was used to estimate the speed of the trace. Beads were excluded if they failed any of the following criteria: power spectral bias < 0.15 over the entire video, power spectral bias < 0.2 over the period analysed for spinning, the integral of the fit Gaussian < 80% of the integral of the subtracted spectrum between $v_0/10$ and the upper of $5v_0$ or the Nyquist frequency, where v_0 was the typical rotation speed for the collected sample (eg $v_0\sim$ 500 Hz was used for wild type at saturating ATP, and hence data was fit between 50 Hz and 2500 Hz; these limits were used to reduce the effect of noise from drift and high collection bandwidth and this condition helps eliminate occurrences of fitting a peak to noise). A symmetry condition was also imposed such that the integral

$$\frac{3}{2\pi}\int_0^{\frac{2\pi}{3}}\left(3d\theta\sum_{j=0}^{2}\left(\frac{N_{\frac{2\pi j}{3}+\theta}^{\frac{2\pi(j+1)}{3}+\theta}}{N_0^{2\pi}}\right)^2\right) \tag{4}$$

was less than 1.5, where N_a^b was the number of data points lying between angles a and b, (this integral lies strictly between 1 and 3, with 1 corresponding to angular data with 3-fold symmetry, and 3 to data with all points at one angle). Typically this integral ranged from 1.00 to 1.1 for normal data; this limit removes data where, for example, the fitting mask has moved between separate beads during the video.

Second, if no pauses were present, angular speed was calculated from the total angular displacement during the video. When pauses were present, the pauses were removed as described below for calculating the kinetics of dwell states. When used as a measure of the true rotation rate of F_1 (e.g. in speed-[ATP] curves), data were rejected if more than 2% of the data points had a radial distance, relative to the ellipse fit, less than one-third the distance to the ellipse. Traces rejected by this criterion tend to have multiple false centre-crossings, and on average produce measured speeds that are lower than the actual rotation speed, as shown in Fig. 2.

Michaelis-menten fits

Michaelis-menten fits (see Fig.s 2,4) were performed using Origin software, via the inbuilt Hill model with cooperativity coefficient fixed to 1 and Levenberg-Marquardt minimisation. Mean

speeds were calculated for each ATP concentration measured (typically selected from 1, 3, 10, 30, 100, 300, 1000 and 3000 μM). Points were weighted according to an estimated standard error of that point; in cases where at least 10 speeds were observed, the conventional estimate for standard error $\left(\frac{s}{\sqrt{n}}\right)$ was used. For points with small numbers of observed speeds (<10), the sample deviation is a noisy estimated of standard deviation, and we instead estimated standard error by assuming the standard deviation to be proportional to speed for all ATP concentrations (this appeared to be a reasonable assumption based on large datasets). For the angle-speed curve in Fig. 2, this assumption was used for all points, and is equivalent to a standard weighting of points by $\left(\frac{1}{\sqrt{n}}\right)$ in log-space.

Comparison between Michaelis Menten and Kinetic fits

Fig. S6 compares the v_{max} values derived from Michaelis-Menten fits to the k_2 and k_3 values derived from kinetic fits at high ATP:Mg (See Table 1). At high ATP:Mg, it is expected that ATP:Mg binding is rapid and the speed of F_1 rotation is limited by the speed of the two ATP:Mg independent processes which occur at the catalytic dwell, that is:

$$\frac{1}{v_{\max}} = 3\left(\frac{1}{k_2} + \frac{1}{k_3}\right). \tag{5}$$

The comparison in Fig. S6 shows near agreement between the two methods, as expected.

References

1. Thompson, R.E., Larson, D.R., Webb, W.W. Precise nanometer localization analysis for individual fluorescent probes. *Biophys J* **82:** 2775-2783 (2002).

2. Okazaki, K., Hummer, G. Phosphate release coupled to rotary motion of F1-ATPase. *Proc Natl Acad Sci* **110:** 16468-16473 (2013).

3. Kim, S.-J., Koh, K., Boyd, S., Gorinevsky, D. l(1) Trend Filtering. *Siam Rev Soc Ind Appl Math* **51:** 339-360 (2009).
